# Molecular Aspects of MAFLD—New Insights on Pathogenesis and Treatment

**DOI:** 10.3390/cimb45110573

**Published:** 2023-11-15

**Authors:** Branka Filipovic, Marija Marjanovic-Haljilji, Dragana Mijac, Snezana Lukic, Suncica Kapor, Slobodan Kapor, Ana Starcevic, Dusan Popovic, Aleksandra Djokovic

**Affiliations:** 1 Department of Gastroenterology, Clinical and Hospital Center “Dr Dragisa Misovic—Dedinje”, Heroja Milana Tepica 1, 11020 Belgrade, Serbia; branka.filipovic3@gmail.com (B.F.); pduschan@gmail.com (D.P.); 2 Faculty of Medicine, University of Belgrade, Dr Subotica Starijeg 8, 11000 Belgrade, Serbia; draganamijac@gmail.com (D.M.); lukic.snezana@gmail.com (S.L.); kaporbg@gmail.com (S.K.); ana.starcevic22@gmail.com (A.S.); drsaska@yahoo.com (A.D.); 3 Clinic of Gastroenterology and Hepatology, Clinical Center of Serbia, Koste Todorovica 2, 11000 Belgrade, Serbia; 4 Department of Hematology, Clinical and Hospital Center “Dr Dragisa Misovic—Dedinje”, Heroja Milana Tepica 1, 11020 Belgrade, Serbia; suncicabjelica@gmail.com; 5 Institute of Anatomy “Niko Miljanic”, Dr Subotica Starijeg 4/2, 11000 Belgrade, Serbia; 6 Department of Cardiology, Clinical and Hospital Center “Bezanijska Kosa”, Dr Zorza Matea s/n, 11080 Belgrade, Serbia

**Keywords:** metabolic-associated fatty liver disease, inflammation, gut microbiota, treatment strategies

## Abstract

Metabolic-associated liver disease (MAFLD) affects up to 70% of overweight and more than 90% of morbidly obese people, and its pathogenesis is rather complex and multifactorial. The criteria for MAFLD include the presence of hepatic steatosis in addition to one of the following three criteria: overweight or obesity, presence of type 2 diabetes mellitus (T2DM), or evidence of metabolic dysregulation. If the specific criteria are present, the diagnosis of MAFLD can be made regardless of alcohol consumption and previous liver disease. The pathophysiological mechanisms of MAFLD, including inflammation, lipotoxicity, mitochondrial disfunction, and oxidative stress, as well as the impact of intestinal gut microbiota, are constantly being elucidated. Treatment strategies that are continually emerging are based on different key points in MAFLD pathogenesis. Yet, the ideal therapeutic option has still not been found and future research is of great importance, as MAFLD represents a multisystemic disease with numerous complications.

## 1. Introduction

**Figure 1:**
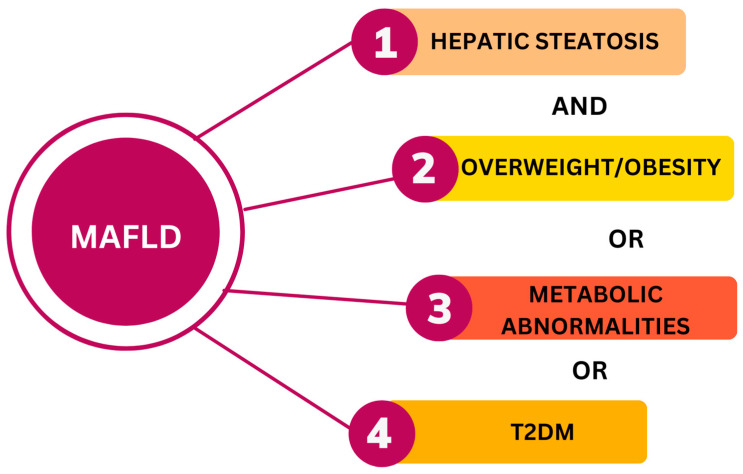
Diagnostic criteria for MAFLD. T2DM—type 2 diabetes mellitus. The figure was created in the Canva program (https://www.canva.com/).

Metabolic-associated fatty liver disease (MAFLD) is the new term related to nonalcoholic fatty liver disease (NAFLD). MAFLD was suggested by experts of The International Consensus Panel of MAFLD in 2020. MAFLD emphasizes the importance of metabolic factors in the development and progression of liver disease. The criteria for MAFLD include the presence of hepatic steatosis in addition to one of the following three criteria: overweight or obesity, presence of type 2 diabetes mellitus (T2DM), or evidence of metabolic dysregulation (Figure 1). Since the association of metabolic syndrome and fatty liver is common, it has been suggested that a new term, MAFLD, is more appropriate. If the specific criteria are present, the diagnosis of MAFLD can be made regardless of alcohol consumption and previous liver disease [1,2].

Hepatic steatosis can be detected using imaging techniques: ultrasound, transient elastography (FibroScan), CT or MR scan, different scores and biomarkers (FIB-4, FLI), and liver biopsy. Overweight/obesity is defined as BMI ≥ 25 kg/m^2^ in Caucasians or BMI ≥ 23 kg/m^2^ in Asians. Metabolic dysfunction implies the presence of at least two metabolic risk abnormalities:

- Waist circumference ≥ 102/88 cm in Caucasian men and women (or ≥90/80 cm in Asian men and women).
- Blood pressure ≥ 130/85 mmHg or specific drug treatment.
- Plasma triglycerides (TG) ≥ 150 mg/dL (≥1.70 mmol/L) or specific drug treatment.
- Plasma HDL-cholesterol < 40 mg/dL (<1.0 mmol/L) for men and <50 mg/dL (<1.3 mmol/L) for women or specific drug treatment.
- Prediabetes (i.e., fasting glucose levels 100 to 125 mg/dL (5.6 to 6.9 mmol/L), 2 h post-load glucose levels 140 to 199 mg/dL (7.8 to 11.0 mmol), or HbA1c 5.7% to 6.4% (39 to 47 mmol/mol)).
- Homeostasis model assessment of insulin resistance (HOMA-IR) score ≥ 2.5.
- Plasma high-sensitivity C-reactive protein level > 2 mg/L [1].

This spectrum of diseases affects up to 70% of overweight and more than 90% of morbidly obese people [3].

## 2. Pathophysiological Mechanisms of MAFLD

**Figure 2:**
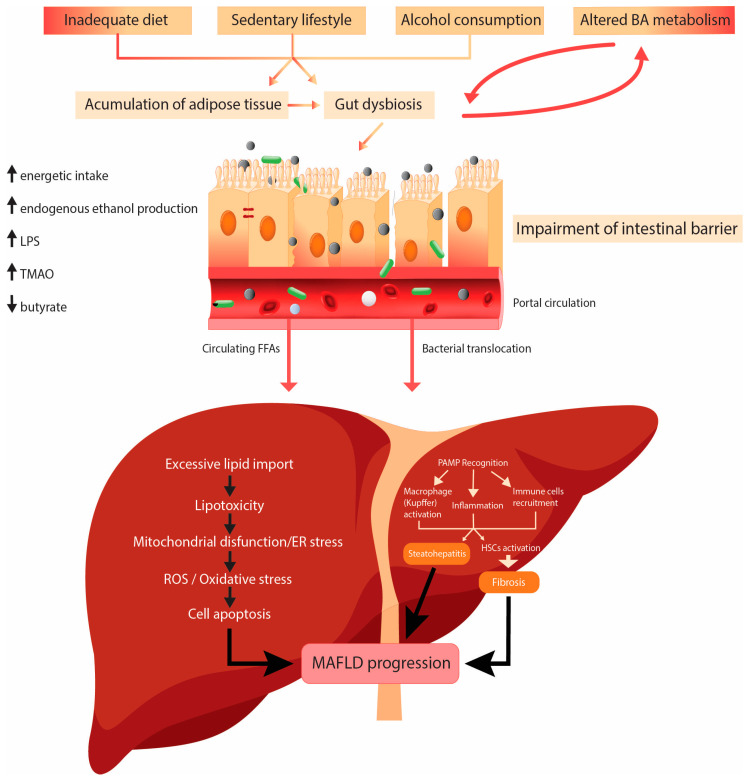
Pathophysiological mechanisms of MAFLD. Abbreviations: LPS—lipopolysaccharide; TMAO—trimethylamine–N-oxide; PAMP—pathogen-associated molecular pattern; ER—endoplasmic reticulum; ROS—reactive oxygen species; HSCs—hepatic stellate cells. The figure was created in Adobe Illustrator.

The pathogenesis of MAFLD is rather complex and multifactorial (Figure 2).

The genetic variations in Patatin-like Phospholipase Domain-containing 3 (PNPLA3), Transmembrane 6 Superfamily Member 2 (TM6SF2), and Membrane Bound O-acyltransferase Domain-containing 7 (MBOAT7) play the principal roles in the development and progression of MAFLD. The PNPLA3 gene is associated with increased hepatic lipid levels and hepatic inflammation, hepatic fibrogenesis, and elevated risk of development of HCC [4]. TM6SF2 is associated with hepatic steatosis and progression of liver disease, because it increases the liver TG content and decreases VLDL secretion [5]. Variants of the MBOAT7 gene are related to alcoholic cirrhosis, but also are associated with the development and severity of MAFLD via remodeling of phospholipids [6].

Environmental factors like high-caloric diet and decreased physical activity play a role in the pathogenesis of MAFLD.

High-fat diet (HFD) means high consumption of fat, typically known as the Western diet. High fat intake plays a significant role in the pathogenesis of MAFLD, and it leads to excessive accumulation of fat in the liver in patients with a genetic predisposition. HFD is very often associated with insulin resistance, lipid disorders, and metabolic and cardiovascular diseases, components of metabolic syndrome. The hypothesis that most likely explains the theory of progression MAFLD to nonalcoholic steatohepatitis (NASH), cirrhosis, and hepatocellular carcinoma is the double-hit hypothesis. The primary disorder contributing to steatosis is IR. IR starts hepatic de novo lipogenesis (DNL) and impaired fatty acid (FA) transport. The second hit implies mitochondrial dysfunction, endoplasmic reticulum stress, inflammation, and many other disturbances [7].

The fatty acids (FAs) in the liver originate from dietary fat, adipose tissue lipolysis, or de novo lipogenesis (DNL) from carbohydrates or other dietary fat precursors. Inside the liver, FAs are esterified into TG and assembled into very-low-density lipoprotein (VLDL) to be secreted in the circulation, oxidized in the mitochondria (β-oxidation), or stored in lipid droplets (LDs). LDs undergo lipid hydrolysis in fasting conditions to provide FAs for β-oxidation. With chronic nutrient overload and IR in MAFLD, the input of FAs to the liver exceeds their disposal via VLDL secretion or β-oxidation [8]. In patients with MAFLD, about 15% of liver FAs originate from the diet, 59% are derived from circulation, and 26% from DNL [9]. IR increases DNL in the liver [10,11]. Two main transcriptional factors are involved in DNL: sterol regulatory element binding protein 1c (SREBP1c), regulated by insulin signaling, and carbohydrate response element binding protein (ChREBP), activated by glucose uptake [12]. The results of mice studies have shown the important role of the peroxisome proliferator-activated receptors (PPARγ) in hepatic lipogenesis [13].

Lipotoxicity is defined as accumulation or transient generation of toxic lipids in LDs (>5% of liver weight), which may result in hepatocyte injury or death. The toxic lipids are the ones that cause cellular injury and cell death in MAFLD pathogenesis. The saturated fatty acids (SFAs) (palmitate, ceramides, lysophosphatidylcholine, and free cholesterol) are directly cytotoxic and can induce progressive liver injury. The monounsaturated free FAs, such as oleate and palmitoleate, may protect from lipotoxicity [14]. The mechanisms of lipotoxicity involve several cellular processes in the liver, such as endoplasmic reticulum (ER) stress, mitochondrial dysfunction, oxidative stress and production of reactive oxygen species (ROS), and intestinal dysbiosis [15].

Mitochondria, ER, and peroxisomes produce ROS in normal cellular metabolism, but 90% of cellular ROS are produced in mitochondria. An HFD increases the amount of substrate available for oxidation and the production of free radicals and damages the antioxidant defense [16]. An imbalance between the production of ROS and the antioxidant activities leads to oxidative stress. Antioxidants with main function to eliminate free radicals involve nonenzymatic molecules such as ascorbic acid (vitamin C), α-tocopherol (vitamin E), glutathione (GSH), carotenoids, and flavonoids and the antioxidant enzymes, such as superoxide dismutase, glutathione peroxidase, and catalase. Excessive ROS production causes mitochondrial damage and activation of Kupffer cells, which then produce cytokines and cause inflammation. ROS activates hepatic stellate cells (HSCs) to produce extracellular matrix, leading to fibrosis [8,16,17].

Mitochondrial dysfunction is a consequence of enormous production of ROS, but in this vicious circle, mitochondrial dysfunction is also the cause of increased production of ROS. Uncontrolled production of ROS in the mitochondria damages mitochondrial components and leads to activation of the mitochondrial quality control (MQC) system. MQC is a specific mechanism responsible for maintaining mitochondrial integrity. This system includes biogenesis, fission, fusion, and mitophagy. Enormous production of ROS can damage structural parts of mitochondria, mitochondrial membrane, DNA, and can activate apoptotic processes, leading to mitochondrial autophagy, known as mitophagy [18,19,20].

A recent study in a “human-like” MAFLD rat model, which was induced by HFD-feeding for 14 weeks in association with thermoneutral housing, showed the effect of HFD on DNA damage and mitochondrial quality control mechanisms in an MAFLD. The results of this study showed that the mtDNA copy number and expression of markers involved in mitochondrial biogenesis were significantly reduced in the HFD rats group. Also, the protein levels of MFN2 (mitofusin 2), GTPase protein, which regulates the process of mitochondria, were significantly reduced in the liver of the HFD rats, but there were no significant changes in the other protein levels of fusion-optic atrophy 1 (OPA1), or in the protein levels of fission-dynamin-related protein 1 (DRP1). These results suggest a disorder of mitochondrial dynamic in MAFLD, probably more of fission. The analysis showed that, in HFD rats, the phosphorylation/activation of AMPK on Thr172 and of unc-51-like kinase 1 (ULK1) on Ser555 were both significantly increased. A significantly increased expression of AMBRA1, binding partners, and downstream effectors of ULK1 confirmed the activation of autophagy in the HFD group. A significant decrease in the expression of key proteins involved in the mitophagy flux in the liver of the HFD rats, PTEN-induced kinase 1(PINK1), the E3 ligase (PARKIN), and microtubule-associated protein 1A/1B-light chain 3B (LC3BII), indicated an impaired autophagic flux [21].

It has been known for a long time that oxidative stress can be assessed using serum marker serum, soluble NOX2-derived peptide, and urine marker, urinary 8-iso-PGF2α. The results of the studies showed that urinary 8-iso-PGF2α and serum-soluble NOX2-derived peptide are increased in patients with nonalcoholic fatty liver. Their level is independent of obesity, diabetes, and metabolic syndrome. Levels of urinary 8-iso-PGF2α and serum-soluble NOX2-derived peptide increase with the severity of liver steatosis at ultrasound. The mentioned study indicated that the markers of oxidative stress can also be used in assessing the severity of liver steatosis [22].

Since ER is involved in both protein and lipid homeostasis, proteotoxic and lipotoxic stress are closely related, and are likely bidirectional. Disequilibrium in the lipid bilayer induces lipotoxic ER stress, while the accumulation of unfolded or misfolded proteins in the ER lumen triggers proteotoxic ER stress. The unfolded protein sensors can be directly activated by toxic lipids. The unfolded protein response (UPR) is activated via the luminal domains of three principal transmembrane sensors: inositol-requiring enzyme (IRE)-1α, protein kinase RNA-like ER kinase (PERK), and activating transcription factor (ATF)-6α. ER is involved in protein folding and trafficking and lipid biosynthesis and trafficking. That is the reason these two types of stress are interconnected. When homeostasis is disturbed and equilibrium cannot be established, ER stress-induced apoptosis occurs [23,24].

## 3. Gut Microbiota—In Sickness and Health

The intestinal microbiota represent a community of more than 200 prevalent bacteria, fungi, and viruses that inhabit the human gastrointestinal tract [25]. During the last decade, constantly emerging research efforts have enlightened us with knowledge of these gut inhabitants, bringing out a different perspective regarding human health. At first, the information of gut microbiota was gathered from the difficult processes of isolating and culturing different species, while later, along with the development of modern methods such as sequence analysis and shotgun metagenomics, the term microbiome emerged, referring to the collective genomes of all microorganisms inhabiting an environment. With these new methods, almost 2000 new bacterial species have been discovered, as well as their authentic functional capacity [26]. This has led to better comprehension of the gut microbiome, yet it remains not fully determined and understood. The human genome consists of about 23,000 genes, whereas the microbiome encodes over three million genes, providing unique metabolic setting and impacting host metabolism in numerous ways [27]. Therefore, the interplay between host and gut microbiota has an important role in pathophysiological mechanisms regarding development of various diseases, such as inflammatory bowel disease (IBD), T2DM, cardiovascular disease (CVD), colorectal cancer (CRC), and MAFLD, which is discussed in this review [28,29]. The diversification of the gut microbiome starts at birth and composition modifications depend on a variety of genetic, nutritional, and environmental factors. Dietary intake is considered as the main culprit for disturbance of the microbiota composition, as well as the bile acid (BA) composition, the alteration of intestinal barrier homeostasis, and activation of inflammatory pathways [30]. Numerous taxonomic studies have tried to characterize the distinctive microbiota signatures correlating with different diseases, including MAFLD [31]. This field of research set microbiota as a potentially important noninvasive biomarker of MAFLD, or main variable for MAFLD prevention or treatment through modulation of microbiota composition. It has been shown that some species are associated with MAFLD, like Proteobacteria, Enterobacteria, Escherichia, or Bacteroides, being of higher abundance in patients with steatohepatitis as compared to the matched healthy controls [32]. Also, a lower abundance of Bacteroidetes, and in particular, the genera Prevotella, has been found in the MAFLD group. Some research demonstrated that the Firmicutes/Bacteroidetes ratio is disturbed in obese patients, as well as in those with MAFLD [33]. Across the MAFLD spectrum from simple steatosis to steatohepatitis and fibrosis, some other distinctive microbial signatures have been established by metagenomic sequencing, for example, an increased abundance of *Escherichia coli* and *Bacteriodes vulgatus* in advanced fibrosis, or an independent association of the genera Ruminococcus with this stage of liver disease [31,34]. Although extensive research has been conducted regarding this issue so far, the results are rather heterogenic and sometimes conflicting, this being probably due to the underlying complex interactions among the different elements and the effect of probable confounders.

In addition to the previously corroborated pathophysiological mechanisms, there is no talk of MAFLD without mentioning the term gut–liver axis, the bidirectional communication between the intestines and the liver. Being more than mere interorgan connection, the gut–liver axis also includes the complex interplay between the immune system and the gut microbiota [35]. There are several entities stretching along the gut–liver axis, with gut microbiota as the main intersection in this interorgan communication. The role of microbiota in MAFLD development and evolution can be substantiated by several mechanisms, which are discussed in the following section.

Intact intestinal barrier plays a crucial role in the preservation of normal environment of the intestinal tract. It represents the first line of defense from unwanted passage of toxins, microorganisms, or their metabolites from gut lumen. Protection strategies include tight junctions (TJs) between adjacent enterocytes, immunoglobulins (sIgA), mucins, antimicrobial lectins, and gut microbiota, being the first soldier in line within organism defense [36]. If any of these components is compromised, the impairment of the intestinal barrier will occur, leading to increased intestinal permeability—the leaky gut syndrome. In addition to being the mere change in composition of commensal bacteria, gut dysbiosis promotes increased intestinal permeability also through changes in expression and distribution of TJs, and a decrease in the secretion of antimicrobial peptides [37,38]. Also, it has been found that gut bacteria can cause alterations of the mucus layer and in that way contribute to the increased intestinal permeability, like *Akkermansia muciniphila,* for example. This Gram-negative bacteria has already been linked to obesity, IR, and MAFLD, and recent studies explored its promising therapeutic potential, as it is shown that oral supplementation with it ameliorates MAFLD and its features via the gut–liver axis [39,40]. In addition to leaky gut, the alteration of vascular barrier has also been explored. These two features, along with the small intestine bacterial overgrowth (SIBO), enable translocation of bacteria or bacterial products from intestinal lumen to portal circulation, representing a sort of warranty of the ensuing step in liver damage—the induction of hepatic inflammatory response to microbial antigens [40,41].

The immune system and its components are the backbone of the gut–liver axis. The gut-associated lymphoid tissue (GALT) consists of intraepithelial lymphocytes, Peyer’s patches, the effector cells in the lamina propria, mesenteric lymph nodes, and isolated lymphoid follicles. There are also immune cells within the liver and the adipose tissue included in the functioning of the gut–liver axis; hence, all these elements normally maintain a balance between tolerance and response to bacterial antigens [42]. In MAFLD, distinct proinflammatory pathways are triggered. In the microbiota-induced inflammation it all starts with bacterial translocation, when pattern recognition receptors (PRRs) recognize pathogen-associated molecular patterns (PAMPs), which are small pathogen components or products, such as lipopolysaccharide (LPS), the major outer membrane part of the Gram-negative bacteria. This LPS endotoxemia and consequent recognition by Toll-like receptors (TLRs) 4 activate an immunologic cascade which is considered to be the pivotal step in the development of inflammation, which distinguishes steatosis from steatohepatitis [43,44]. Numerous studies have shown elevated serum levels of LPS in patients with steatohepatitis, compared to the ones with simple steatosis or healthy subjects, thus confirming the role of gut dysbiosis in metabolic-associated steatohepatitis (MASH) development [44,45]. Also, recent studies confirmed the important role of LPS-TLR4 signaling in MAFLD pathogenesis, where the treatment with sevelamer, a BA sequestrant, was conducted. Sevelamer was binding to LPS in the intestinal lumen, promoting its fecal excretion, leading to restoration of the TJs and reduction of portal LPS levels, thus suppressing the hepatic TLR4 signaling pathway. In addition, this treatment improved the diversity of gut microbiota by decreasing the abundance of MAFLD-promoting species [46].

Though LPS-mediated inflammation via TLR4 may be the primary driver of the immune response in MAFLD, it is not the only one. The inflammation cascade can also be triggered through TLR9, TLR5, or TLR2, whose ligands are methylated DNA, flagellins, and peptidoglycan from Gram-positive bacteria (in the order given) [47]. When stimulated, TLR4 generates intracellular signaling pathways, specifically NF-κB and MAPK, which further induce the synthesis of proinflammatory cytokines like TNF [48]. Kupffer cells are the main culprit for liver injury as they have the largest number of expressed TLR4, along with the ability to produce ROS and contribute to oxidative stress. In addition to induction of oxidative stress and inflammation, fibrogenesis is also induced in this scenario, by triggered HSCs [49].

The intestinal microbiota has a significant influence on host metabolic state, not only by facilitating harvest of nutrients from food, but also by its capacity to produce certain metabolites, thus being the possible key regulator of the host metabolic profile. These functional interactions are primarily defined through metabolism of polysaccharides, i.e., microbial fermentation of dietary indigestible fibers that produce short chain fatty acids (SCFAs)—acetate, propionate, and butyrate [50]. Emerging evidence strongly suggests that SCFAs not only have a say in MAFLD pathogenesis via hepatic glucose and lipid homeostasis but are speculated to play a key role in the gut–brain axis [51,52]. The positive effects of a high-fiber diet are already very well known. Studies have shown that butyrate represents the most potent anti-inflammatory mediator, which can also promote tight junction function and intestinal integrity. SCFAs accomplish their functions by binding to the specific G-protein-coupled receptors (GPRs) or through the activation of PPARs [53,54].

Along with playing a key role in SCFAs metabolism, it has been demonstrated that gut microbiota are strongly connected to the metabolism of BA, because of their capacity to process deconjugated BAs into secondary ones and, in that way, promote their fecal excretion [55,56]. Alterations of the BA metabolism lead to the suppressed activity of BA receptors FXR and TGR5, which further leads to decreased energy outflow and increased lipogenesis, intensified BA synthesis, and increased macrophage activity [54,57].

Except for meddling in BA metabolism, it has been known that intestinal bacteria are involved in choline metabolism. Choline, derived from diet, plays an important role in liver and brain functioning. It is transformed into lecithin which helps excretion of VLDL from liver, thus preventing hepatic lipid accumulation [58]. Gut bacteria produce enzymes that convert choline into toxic molecules, like trimethylamine (TMA), which then travel through portal circulation to the liver where they are transformed into TMA-N-oxide (TMAO). TMAO can also be derived from dietary L-carnitine. This toxic compound has been linked not only to hepatic damage but also to cardiometabolic complications and mental disorders [59,60,61,62]. Regarding liver diseases, research has shown that intestinal dysbiosis can promote steatosis and chronic inflammatory status, as seen in MASH, by reducing choline levels and increasing levels of TMAO [63]. Also, it has been demonstrated that some ethanol-producing microbial strains might be involved in the pathogenesis of MAFLD, like high-alcohol-producing *Klebsiella pneumoniae* [64]. Both nonalcoholic and alcoholic liver disease show increased levels of ethanol and its metabolites, acetaldehyde and acetate, which have been linked to hepatic injury by causing mitochondrial dysfunction and inflammation [65].

## 4. The Impact of Intestinal Microbiota on Cardiometabolic Function

Now, it is well known that MAFLD should be referred to as a multisystemic disease, as it has been linked to CVD, chronic kidney disease, and some brain disorders [66]. With MAFLD and CVD being highly prevalent, and both being associated with metabolic alterations, they are often found in coexistence. The answer to this occurrence may lie in the mutual pathophysiological pathways comprising oxidative stress, persistent low-grade inflammation, and IR [67]. Along with that, the answer is maybe hiding in the disturbed intestinal microbiota and its capacity to interfere with host metabolism, or its production of toxic metabolites [68]. Several meta-analyses and cohort studies showed that patients with MAFLD are at higher risk of major adverse cardiovascular events (MACEs), independently of the extent of coronary disease or other CV risk factors. The previously mentioned gut-microbiota-derived TMAO has been established as a risk factor of CVD, as its high levels are closely related to the occurrence of atherosclerosis, heart failure, hypertension, and other CVDs [69,70]. The mechanisms of action in the pathogenesis of atherosclerotic disease and other CV entities include accumulation of cholesterol, endothelial dysfunction, and activation of proinflammatory and prothrombotic pathways [71,72].

With regard to MAFLD undoubtedly being connected to CVD, it is with great certainty that we can now establish causal connection between these two clinical entities, with intestinal microbiota being the possible underlying common denominator. It is of crucial importance that patients with MAFLD undergo cardiovascular assessment. As numerous efforts are still being made in order to elucidate more precisely this cobweb of correlations, new promising therapeutic targets are emerging for CVD along with MAFLD.

## 5. Treatment Options of NAFLD/MAFLD

In the last 15 years, numerous different molecules have been tested for the treatment of NAFLD/MAFLD. Since then, the goal of NASH/NAFLD therapy has been changed, and today includes three major pharmacological targets: the reduction of liver steatosis, the reduction of inflammation, and the regression of fibrosis [73]. The first approach to NASH treatment was dietary changes together with lifestyle changes, from a sedentary to a physically active lifestyle [74]. Nowadays, it is well known that NASH is a part of complex metabolic diseases with multiplex pathophysiology pathways including complicated genetic background together with different environmental factors [75,76]. On the other hand, a wide spectrum of disease progression, from mild inflammation in fatty liver to the end-stage liver disease with cirrhosis and its complications, contributes to the complexity of pharmacological interventions requiring specific interventions for different stages of disease [77]. For these reasons, the pharmacotherapy for this heterogeneous disease includes a variety of molecules with different pharmacological targets, from drugs targeting metabolic conditions that contribute to development and progression of NASH (antidiabetic and hypolipidemic agents) to drugs used to treat liver inflammation and liver fibrosis.

Thus far, more than 20 drugs have been tested for the treatment of NASH, while many molecules are currently in the development or in preclinical trials.

In this review, we discuss the molecules in the most advanced clinical development, including hypolipidemic and antidiabetic drugs, PPAR agonists, FXR agonists, FGF analogs, and thyroid hormone receptor (THR) agonists. Table 1 shows some of the results of different clinical trials that are in different phases, active or terminated, in the treatment of NASH/MASH (Table 1) [73,78].

As previously mentioned, NAFLD is associated with metabolic comorbidities including T2DM, hypertriglyceridemia, hypercholesterolemia, IR, hypertension, and obesity or overweight in some patients. Hypolipidemic and antidiabetic drugs are well-studied medical agents aiming to stimulate metabolic processes that cause NAFLD and to reduce intrahepatic fat accumulation. Treatment with statins and ezetimibe aims to reduce circulating and tissue-accumulated cholesterol and is recommended in NAFLD. These molecules may improve the lipid profile in NAFLD [79]. Proprotein convertase subtilisin/kexin type 9 (PCSK9) is an important player in cholesterol homeostasis and intracellular lipogenesis, with a possible impact of circulating PCSK9 in the early stages of NASH, but not in the late stage of liver disease. Up to now, data from clinical trials with PCSK9 inhibitors reassure the safety of these molecules. However, real lifelong term data are required [80,81].

The efficacy of several antidiabetic drugs has been studied in NAFLD. Pioglitazone showed histological improvement, while the effect on liver fibrosis was not significant in both study groups: patients with T2DM and patients with IR [82,83]. Sodium-glucose co-transporter 1/2 (SGLT1/2) inhibitors and GLP-1RAs (glucagon-like peptide 1 receptor agonists) are newer antidiabetic agents that reached late-stage clinical development in NASH. Dapagliflozin is an oral SGLT2 inhibitor which impedes glucose reabsorption in proximal tubule, with consecutive glucosuria and plasma glucose reduction. Dapagliflozin is most likely to act through the reduction of visceral fat and improvement in liver tests and metabolic variables in patients with T2DM and NASH [84]. Other SGLT1/2 inhibitors are in phase 2 studies. The ELIVATE study is assessing licogliflozin alone or in combination with tropifexor, an agonist of the BA receptor FXR that may improve fibrosis [85]. Another ongoing study is evaluating a synthetic FXR agonist MET409 alone or combined with empagliflozin in patients with NASH and T2DM [86]. Semaglutide (GLP-1RAs) mimics GLP-1, a gut hormone released in response to eating and that reacts to release insulin, and also interacts with the brain to reduce appetite. The FDA (US Food and Drug Administration) has approved semaglutide in 2021 for adults with obesity or overweight with at least one condition: hypertension, T2DM, or high cholesterol.

A phase 3 clinical study with GLP-1RA (ESSENCE) evaluates the potential resolution of NASH and improvement in fibrosis in noncirrhotic patients with NAFLD [87]. A phase 2 trial evaluated the effect of liraglutide (1.8 mg daily) in patients with NASH, while preliminary data confirmed histological resolution of NASH (39% with liraglutide vs. 9% in placebo group) [88]. Modified GLP1 and GIP peptides such as cotadutide, tirzepatide, and efinopegdutide are in early clinical trials and results will be published soon [89,90].

Peroxisome proliferator-activated receptors (PPARs) are ligand-activated transcription factors of the nuclear hormone receptor family comprising three subtypes: PPARα, PPARγ, and PPAR β/δ. PPARs are mainly located in the liver, brown adipose tissue, and macrophages. PPARs activate FA oxidation, lower synthesis of triglycerides, and increase insulin sensitivity; thus, PPAR agonists modulate key metabolic, inflammatory, and fibrogenic pathways in the pathogenesis of NAFLD [91]. PPAR agonists have been approved for patients with T2DM and NASH confirmed by liver biopsy [92]. However, there are still ongoing clinical trials to assess the efficacy of PPAR agonists in patients with NASH without T2DM. Lanifibranor, the only pan-PPAR agonist, has been assessed in noncirrhotic biopsy-confirmed high-active NASH patients in a phase 2 trial with promising findings that support further assessment of lanifibranor in phase 3 trials [93]. Pemafibrate is a novel selective PPARα modulator (SPPARMα) investigated in a phase 2 trial in high-risk NAFLD patients. Preliminary results show that pemafibrate may be a promising therapeutic agent for NAFLD and a promising candidate for combination therapy with agents that reduce liver fat content [94].

Eight years ago, the FLINT study demonstrated for the first time that obeticholic acid (OCA) can improve hepatic lipid and glucose metabolism and, through its anti-inflammatory and antifibrotic activity, improve liver histopathology and fibrosis in NASH. Furthermore, it was shown that OCA increases insulin sensitivity and improves glucose homeostasis in patients with NASH and T2DM. However, it was not clear whether these effects were drug (OCA)- or target (FXR)-specific [95]. In addition to BA synthesis, this receptor is involved in glucose and lipid metabolism, and in the regulation of inflammation. Up to now, many studies have demonstrated that FXR modulation may have various implications in the treatment of NASH. Emerging data have demonstrated that FXR agonists might be quite effective in reducing different sequelae associated with NASH and even different dysfunctional processes coexisting with liver cirrhosis and its complications. However, important manifestations of FXR agonists, both steroidal and nonsteroidal, are pruritus and there is worsening of the HDL-c/LDL-c ratio, since all investigated FXR agonists demonstrated these effects in different trials in humans [96]. Novel agents like MET642 and MET409 are structurally optimized synthetic nonbile acid FXR agonists with enhanced potency. Preliminary results have shown that these molecules produce less pruritus than OCA, but complete clinical results are expected in the future. Ciloflexor is a small-molecule nonsteroidal agonist of FXR that showed efficacy in improving liver steatosis and serum bile acids in patients with NASH [97]. The preliminary results of the phase 2 clinical study demonstrated synergistic activity of ciloflexor in combination with firsocostat (acetyl-CoA carboxylase inhibitor) and semaglutide, in decrease of liver steatosis and improvement of liver biochemistry [98].

It is well known that the thyroid hormones significantly influence energy metabolism, lipid utilization, and glucose homeostasis. Numerous trials have shown an inverse correlation between serum TH levels and NAFLD, given that patients with hypothyroidism have increased risk for NAFLD and vice versa [99]. Moreover, thyroid hormones (THs) stimulate FA β-oxidation and oxidative phosphorylation in the liver. Pharmacological control of these pathways would likely impact the treatment of metabolic syndrome and MAFLD. Because of that, experimental and clinical trials have provided evidence of potential utility of the activation of T3-dependent pathways in MAFLD, and even that some metabolites of thyroid hormones (THs) could also play a role in the treatment of metabolic syndrome. THs exert their physiological effects by binding to the thyroid hormone receptors (THRs) α and β. The β isoform is the major THR expressed in the liver, while the α isoform is specifically abundant in the heart. This observation has led to the development of several THRβ-agonists and organ-selective (liver-selective) thyromimetics in the last 25 years [100]. Recently, novel thyromimetics that target liver and/or main TH receptor (THR) isoform in the liver, THR-β, have been developed to treat NAFLD. These promising agents have fewer thyrotoxic effects, such as arrhythmias and osteoporosis, due to their selectivity for the THR-β [101]. Liver-directed THR-β agonists, VK2879 and MGL-3196, have been studied for their effects on liver steatosis, inflammation, and fibrosis in NAFLD. Preliminary assessment showed improved liver steatosis and liver fibrosis in patients with NAFLD in comparison to placebo [102]. In a phase 3 trial, the THR-β agonist resmetirom improved lipid metabolism and liver biochemistry and decreased liver steatosis in patients with NAFLD, while there were no major safety concerns. A phase 3 study that includes patients with NASH and fibrosis is still ongoing and results will be available soon [103].

Currently, many new molecules with antifibrotic and anti-inflammatory effects have been studied regarding NAFLD treatment. Many of them are in the early stage of clinical development, while agents that are already in phase 2 or phase 3 of clinical trials are cenicriviroc (CVC), a small molecule antagonist that blocks chemokine 2 and 5 receptors, both with important roles in liver inflammation and fibrosis, in combination with tropifexor [104]. Balapectin is a complex carbohydrate that targets galectin-3, which plays a role in inflammatory response and fibrosis. A phase 2b/3 trial is analyzing the effect of belapectin in patients with NAFLD in the stage of compensated cirrhosis and portal hypertension, compared to placebo [105].

Endocrine fibroblast growth factor (FGF) analogs have emerged as promising agents due to their ability to act in two directions: directly on the liver, and to improve metabolic processes in the whole organism to a healthier state. The subfamily of FGFs, consisting of FGF19 and FGF21, may decrease liver steatosis and hepatocyte injury and consecutively suppress inflammation and liver fibrosis. Available data from clinical and preclinical studies with FGF19 and FGF21 analogs in NASH and underlying metabolic diseases showed improvement in liver fibrosis, inflammation, and liver steatosis, and improvement in liver biochemistry. The safety profile has been favorable so far [106,107,108,109].

## 6. Conclusions

The pharmacological scenario of MAFLD/NAFLD therapy will certainly change in the next future. The results of the latest clinical trials show that the three major components of MAFLD—steatosis, inflammation, and fibrosis—can be pharmacologically targeted. Although therapies on trial have acceptable tolerance and a good safety profile, a drug with the potential to ameliorate all components of MAFLD is yet to be identified. Indeed, the results of single-drug trials, most of them preliminary, showed improvement of MAFLD components in only a proportion of patients, in general lower than 50%. Combination therapy looks like a promising strategy. The rationale behind the drug combination is, on one hand, to increase the efficacy of one single drug and, on the other hand, to reduce the side effects of one drug by allowing the use of a lower dose, or by controlling the side effects of the first drug. Therefore, the best treatment will probably be defined in the next few years, not only according to different disease stages but also aiming to tailor the treatment for each patient according to the presence of comorbidities.

## Figures and Tables

**Table 1:** Clinical trials in NASH/MASH.

Molecule	Mechanism of Action	Primary Objective	Results
New antidiabetic agents			
Semaglutide	Agonist GLP1	NASH resolution without worsening of fibrosis	NASH resolution No fibrosis improvement
Liraglutide	Agonist GLP1	Histological resolution of NASH	NASH resolution with worsening of fibrosis
Dapaglifozin	Inhibitor of SGLT2	Improvement in scored liver	NA
PPAR modulators			
Pioglitazone	PPARγ agonist	NASH resolution without worsening of fibrosis	NA
Pioglitazone, vitamin E	PPARγ agonist	Improvement in scored liver histology	Vitamin E vs. placebo: improvement Pioglitazone vs. placebo: no improvement
Pemafibrate	PPARα agonist	Percentage change in liver fat content measured by MRI from baseline to week 24	No differences in liver fat content
Saroglitazar (oral)	Dual PPARα and PPARγ agonist	Reduce ALT from baseline	Improvement of NASH by liver biopsy after 52 weeks
Elafibranor(oral) PPARα/γ/δ agonists	Dual PPARα and PPARδ agonist	NASH resolution	No improvement in NASH
Lanifibranor	Pan-PPAR agonist	Histological resolution of NASH	Improvement of NASH and fibrosis by liver biopsy
FXF agonists	CCR2/CCR5 agonist	Improvement in liver fibrosis by ≥1 stage and no worsening of steatohepatitis on liver histology	Ended trial by interymanalisys and lack of efficacy
PXL065	Inhibitor of mitochondrial pyruvate carrier and acyl-CoA synthetase 4	Hepatic fat fraction measured by MRI	Improvement in liver fat content and improve in fibrosis stage
Aramchol	Partial inhibitor of hepatic stearoyl-CoA desaturase	Hepatic fat fraction measured by MRI	No changes in liver fat by MRS. Improvement in liver fibrosis by ≥ 1 stage and no worsening of NASH on liver histology
Selonsertib	Inhibitor of ASK1	≥1-stage improvement in fibrosis according to the NASH CRN classification without worsening of NASH at week 48	Study failed

## Abbreviations

**Acronym**	**Full Terminology**
AMBRA1	Activating molecule in Beclin1-regulated autophagy
AMP	Adenosine monophosphate
AMPK	AMP-activated protein kinase
ATF	Activating transcription factor
BA	Bile acid
BMI	Body mass index
ChREBP	Carbohydrate response element binding protein
CRC	Colorectal cancer
CVC	Cenicriviroc
CVD	Cardiovascular disease
DNA	Deoxyribonucleic acid
DNL	De novo lipogenesis
DRP1	Fission-dynamin-related protein 1
ER	Endoplasmic reticulum
FA	Fatty acid
FDA	US Food and Drug Administration
FGF	Fibroblast growth factor
FXR	Farnesoid X receptor
GALT	Gut-associated lymphoid tissue
GLP	Glucagon-like peptide
GLP-1RAs	Glucagon-like peptide 1 receptor agonists
GPRs	G-protein-coupled receptors
GSH	Glutathione
GTP	Guanosine triphosphate
HCC	Hepatocellular carcinoma
HFD	High fat diet
HOMA-IR	Homeostasis model assessment of insulin resistance
HSCs	Hepatic stellate cells
IBD	Inflammatory bowel disease
Ig	Immunoglobulin
IR	Insulin resistance
IRE	Inositol-requiring enzyme
LC3BII	Microtubule-associated protein 1A/1B-light chain 3B
LDs	Lipid droplets
LPS	Lipopolysaccharide
MACE	Major adverse cardiovascular events
MAFLD	Metabolic-associated liver disease
MASH	Metabolic-associated steatohepatitis
MBOAT7	Membrane Bound O-acyltransferase Domain-containing 7
MFN2	Mitofusin 2
MQC	Mitochondrial quality control
NADPH	Nicotinamide Adenine Dinucleotide Phosphate
NAFLD	Nonalcoholic fatty liver disease
NASH	Nonalcoholic steatohepatitis
NOX	NADPH oxidase
OCA	Obeticholic acid
OPA1	Fusion-optic atrophy 1
PAMPs	Pathogen-associated molecular patterns
PARKIN	E3 ligase
PCSK9	Proprotein convertase subtilisin/kexin type 9
PERK	Protein kinase RNA-like ER kinase
PG	Prostaglandin
PINK1	PTEN-induced kinase 1
PNPLA3	Patatin-like Phospholipase Domain-containing 3
PPAR	Peroxisome proliferator-activated receptors
PPRs	Pattern recognition receptors
RNA	Ribonucleic acid
ROS	Reactive oxygen species
SCFAs	Short chain fatty acids
SFAs	Saturated fatty acids
SGLT1/2	Sodium-glucose co-transporter 1/2
SIBO	Small intestine bacterial overgrowth
SPPARMα	Selective PPARα modulator
SREBP1c	Sterol regulatory element binding protein 1c
T2DM	Type 2 diabetes mellitus
TG	Triglycerides
TH	Thyroid hormones
THR	Thyroid hormone receptor
THR	Thyroid hormone receptors
TJs	Tight junctions
TLRs	Toll-like receptors
TM6SF2	Transmembrane 6 Superfamily Member 2
TMA	Trimethylamine
TMAO	Trimethylamine—oxide
ULK1	Unc-51-like kinase 1
UPR	Unfolded protein response
VLDL	Very-low-density lipoprotein
